# Isolated nonsyndromic cleft palate: multicenter epidemiological study in the Brazil

**DOI:** 10.1186/s12903-023-03197-3

**Published:** 2023-07-14

**Authors:** Samuel Trezena, Renato Assis Machado, Silvia Regina de Almeida Reis, Rafaela Scariot, Ana Lúcia Carrinho Ayroza Rangel, Fabrício Emanuel Soares de Oliveira, Anna Júlia Borges, Alissa Tamara Silva, Daniella R. Barbosa Martelli, Hercílio Martelli Júnior

**Affiliations:** 1 Postgraduate Program in Primary Health Care, State University of Montes Claros, UNIMONTES, Prof. Darcy Ribeiro University Campus, Prof. Rui Braga Avenue, Vila Mauricéia, Montes Claros, MG Postal Code: 39401-089 Brazil; 2 Department of Oral Diagnosis, School of Dentistry, University of Campinas (FOP/UNICAMP), Piracicaba, São Paulo Brazil; 3 Hospital for Rehabilitation of Craniofacial Anomalies, University of São Paulo, Bauru, São Paulo Brazil; 4 Graduate Program in Oral Biology, School of Dentistry, University of Campinas, Piracicaba, São Paulo Brazil; 5 Department of Basic Science, Bahiana School of Medicine and Public Health, Salvador, Bahia Brazil; 6 Department of Oral and Maxillofacial Surgery, School of Health Science, Federal University of Paraná (UFPR), Curitiba, Brazil; 7 Center of Biological Sciences and of the Health, School of Dentistry, State University of Western Paraná, Cascavel, Paraná Brazil; 8 Center for Rehabilitation of Craniofacial Anomalies, Dental School, University of José Rosário Vellano, Alfenas, Minas Gerais Brazil; 9 Department of Oral Diagnosis, Dental School, State University of Montes Claros, UNIMONTES, Montes Claros, Minas Gerais Brazil

**Keywords:** Isolated cleft palate, Epidemiology, Oral clefts

## Abstract

**Background:**

Nonsyndromic orofacial clefts (NSOC) are the craniofacial most common congenital malformations. There are evidences that the nonsyndromic cleft palate (NSCP) development differs from other NSOC. However, most of the publications treat NSCP without considering that information. Furthermore, few studies focus on NSCP. The aim of this study was to describe epidemiological findings of patients with isolated NSCP in Brazil.

**Methods:**

In this cross-sectional multicenter study, four reference Centers for treatment in three different Brazilian states was investigated. Data were obtained from clinical records of patients, between November 2021 and June 2022. Researched variables were sociodemographic, clinical characteristics and pregnancy and family history. Pearson’s chi-square and ANOVA One-way tests were used for associations.

**Results:**

Majority were female (58.1%), white (60.7%) with incomplete NSCP (61.2%). There was an association between complete NSCP and a positive history of medical problems during pregnancy (*p* = 0.016; 27.9%; OR: 1.94; 1.12–3.35). Systemic alterations were perceived in 40.6% of the sample with odds ratio for development of the complete type (OR: 1.21; 0.74–1.97). Higher OR was visualized in medication use during pregnancy (OR: 1.35; 0.76–2.37) and positive family history of oral cleft (OR: 1.44; 0.80–2.55). Dental and surgical care was associated with higher age groups (*p* < 0.050).

**Conclusions:**

NSCP was most prevalent in white skin color female. Complete NSCP is associated with medical problems during pregnancy. Medication use during pregnancy and positive family history of oral cleft increase the chance of developing complete NSCP.

## Background

Nonsyndromic orofacial clefts (NSOC) are the most common congenital malformations in the craniofacial region with prevalence to about 1 affected in every 700 live births [1]. Its etiology is multifactorial and complex due to its genetics component related to a wide variety of candidate genes combined with environmental risk factors [1–3], as associations are the medications use [4, 5] and pregnancy diseases [6], maternal smoking habit [7], folic acid deficiency [8], exposure to environmental pollution [9], history of stillbirth and ethnic variations [10]. NSOC can be broadly classified in cleft lip (NSCL) and cleft palate only (NSCP), or both cleft lip and palate (NSCLP) and rare facial clefts [11].

The most of publications treat the NSCP without considering that their development is different than the NSCLP and NSCL [12]. Evidences that the NSCP development differs from other NSOC that are related by the metabolic signals responsible for palate closing that synthesizes different molecular markers present in NSCL and NSCLP [11, 13]. In addition, the palate development after 8th weeks, while the lip stared around the 4th week of intrauterine life [3].

Some results associates the NSCP presences with syndromes, malformations and the female [7, 9, 14−18], presenting possible phenotypes characteristic of the manifestation of this anomaly. Furthermore the NSCP are commonly seen in patients who are more vulnerable and who will generally require greater increased multidisciplinary care [18]. Although there are numerous studies that evaluate the associated factors and the epidemiology of NSOC, few of them focus on NSCP [19, 5]. Thus, the aim of this study was to describe epidemiological findings of patients with isolated NSCP in Brazil.

## Methods

### Study design and participants

Cross-sectional multicenter study, carried out in three different Brazilian states. Data were obtained from clinical records of patients, between November 2021 and June 2022, from four reference Centers for treatment in the Brazil: [1] Center for the Rehabilitation of Craniofacial Anomalies, University of José do Rosário Vellano, Alfenas, Minas Gerais; [2] Santo Antonio Hospital, Salvador, Bahia; [3] Association of Carriers of Cleft Lip and Palate – APOFILAB, Cascavel, Paraná; and [4] Center for Integral Assistance to the Cleft Lip Palatal, Curitiba, Paraná.

### Data collection

The variables collected were: sex (male/female); cleft palate type (incomplete/complete); age; geographic region (Brazilian states: Bahia/Paraná/Minas Gerais); skin color (white/non-white); medication use during pregnancy (yes/no); medical problems during pregnancy (yes/no); family history of cancer (yes/no); family history of oral cleft (yes/no); underwent surgery (yes/no); number of surgeries undergone; underwent orthognathic surgery (yes/no); dental care (yes/no) and systemic alterations (yes/no).

### Data analyses

Data were analyzed using Statistical Package for the Social Sciences (SPSS) version 22.0 and the results were presented as absolute numbers (n) and as frequency (%). Continuous numeric variables were presented mean and standard deviation (SD) values. Pearson’s chi-square and ANOVA One-way tests were used for associations. All tests were conducted with 95% set as the significance level.

## Results

A total of 313 patients with NSCP, predominantly female (58.1%) and white skin color (60.7%) participated in the study. NSCP incomplete was most common (61.2%) (Table 1).

**Table 1:** Characteristics of patients with nonsyndromic cleft palate (NSCP) according to three different Brazilian states

	Total	Bahia	Paraná	Minas Gerais
	n (%)	n (%)	n (%)	n (%)
**Sex**				
Male	131 (41.9)	55 (39.0)	27 (45.0)	49 (43.8)
Female	182 (58.1)	86 (61.0)	33 (55.0)	63 (56.2)
**Cleft palate type**				
Incomplete cleft palate	188 (61.2)	76 (53.9)	30 (54.5)	82 (73.9)
Complete cleft palate	119 (38.8)	65 (46.1)	25 (45.5)	29 (26.1)
**Age**	13.3 (± 13.4)	20.6 (± 10.2)	10.2 (± 8.9)	5.9 (± 14.2)
**Skin color**				
White	184 (60.7)	32 (23.9)	55 (91.7)	97 (89.0)
Non-white	119 (39.3)	102 (76.1)	05 (8.3)	12 (11.0)
**Medication use during pregnancy**				
Yes	79 (34.3)	39 (33.3)	18 (66.7)	22 (25.6)
No	151 (65.7)	78 (66.7)	09 (33.3)	64 (74.4)
**Medical problems during pregnancy**				
Yes	159 (62.8)	103 (78.6)	31 (83.8)	25 (29.4)
No	94 (37.2)	28 (21.4)	06 (16.2)	60 (70.6)
**Family history of cancer**				
Yes	94 (35.9)	54 (39.7)	31 (91.2)	09 (9.8)
No	168 (64.1)	82 (60.3)	03 (8.8)	83 (90.2)
**Family history of oral cleft**				
Yes	64 (25.4)	36 (26.1)	18 (72.0)	10 (11.2)
No	188 (74.6)	102 (73.9)	07 (28.0)	79 (88.8)
**Underwent surgery**				
Yes	212 (67.9)	110 (78.0)	51 (86.4)	51 (45.5)
No	100 (32.1)	31 (22.0)	08 (13.6)	61 (54.5)
**Number of surgeries undergone**	1.53 (± 0.9)	1.5 (± 0.8)	1.8 (± 1.3)	1.3 (± 0.7)
**Underwent orthognathic surgery**				
Yes	46 (17.1)	34 (24.3)	03 (16.7)	09 (8.1)
No	223 (82.9)	106 (75.7)	15 (83.3)	102 (91.9)
**Dental care**				
Yes	194 (64.7)	128 (92.1)	46 (90.2)	20 (18.2)
No	106 (35.3)	11 (7.9)	05 (9.8)	90 (81.8)
**Systemic alterations**				
Yes	115 (40.6)	53 (37.6)	22 (62.9)	40 (37.4)
No	168 (59.4)	88 (62.4)	13 (37.1)	67 (62.7)

It was found that individuals whose mothers reported having had medical problems during pregnancy (35.1%) were 1.94 more likely to have incomplete NSCP (*p* = 0.016). There is a slight tendency to develop complete NSCP in children with a history of NSOC in the family, whose mother used some type of medication during pregnancy and present with some kind of systemic alteration. However, no statistically significant association was found (*p* > 0,005) (Table 2).

**Table 2:** Characteristics of the patients and clinical history according cleft palate type

	Cleft Palate Type			
	Incomplete n (%)	Complete n (%)	OR (IC_95%_)	*p-*value
**Sex**				
Female	108 (35.2)	69 (22.5)	1.02 (0.64–1.62)	0.926
Male	80 (26.1)	50 (16.3)	-	
**Skin color**				
White	113 (38.0)	65 (21.9)	1.30 (0.81–2.09)	0.272
Non-white	68 (22.9)	51 (17.2)	-	
**Medication use during pregnancy**				
Yes	46 (20.2)	32 (14.0)	1.35 (0.76–2.37)	0.296
No	99 (43.4)	51 (22.4)	-	
**Medical problems during pregnancy**				
Yes	88 (35.1)	70 (27.9)	1.94 (1.12–3.35)	0.016*
No	66 (26.3)	27 (10.8)	-	
**Family history of cancer**				
Yes	59 (22.7)	34 (13.1)	1.03 (0.60–1.74)	0.919
No	107 (41.2)	60 (23.1)	-	
**Family history of oral cleft**				
Yes	35 (14.0)	28 (11.2)	1.44 (0.80–2.55)	0.223
No	120 (48.0)	67 (26.8)	-	
**Underwent surgery**				
No	64 (20.9)	33 (10.8)	1.36 (0.82–2.24)	0.234
Yes	123 (40.2)	86 (28.1)	-	
**Dental care**				
No	79 (26.8)	26 (8.8)	2.70 (1.58–4.53)	< 0.001*
Yes	101 (34.2)	89 (30.2)	-	
**Systemic alterations**				
Yes	65 (23.3)	46 (16.5)	1.21 (0.74–1.97)	0.446
No	106 (38.0)	62 (22.2)	-	

Statistical Significance: *Chi-square test

Table 3 shows that even at a more advanced age there are patients who have not yet undergone surgical or dental care. Patients who had undergone more than one surgical procedure were more likely to be in a higher age group. (*p* = 0.002).

**Table 3:** Surgery submitted and dental care by age of patients with nonsyndromic cleft palate (NSCP).

	Underwent surgery	p-value	Dental care	p-value	Number of surgeries undergone		p-value
Age Group	n (%)		n (%)		n	Mean**	
≤ 01	31 (14.8)		13 (6.8)		34	1.1	
02–05	16 (7.6)		11 (5.7)		12	1.0	
06–10	15 (7.1)	< 0.001*	20 (10.4)	< 0.001*	15	1.0	0.002***
11–17	61 (29.0)		65 (33.9)		91	1.6	
≥ 18	87 (41.4)		83 (43.2)		149	1.7	
Total	210 (100.0)		192 (100.0)		301	1.5	

Statistical Significance: *Chi-square test. **Per individual mean. ***ANOVA one-way test

## Discussion

It is important to highlight that until the writing of this article only two studies [14, 15] have identified possible factors associated with the development of NSCP typesHowever, unlike another Brazilian study [15], complete NSCP was more prevalent in our study.

Investigating the epidemiological features from most studies showed a predilection for NSCL/P in males and NSCP in females, in Brazil and in the world [9, 12, 14−17]. Most of the participants in this study were female, in line with the findings of previous studies. The relationship between NSCP and the female sex can be related to the presence of sex hormones in the increase in cleft, relationship with the x chromosome and mainly due to the fact that the palate in female babies closes a week later than in male babies [19].

Skin color tends to be predominantly whiteas well as in most publications [13, 20−22]. However, the miscegenation of ethnicities and races is a factor that impacts these results of surveys with NSCL/P individuals [23].

Studies have attempted to understand the differences in cleft subtypes in relation to social, demographics and gestational history variables [4, 14, 15]. We found that individuals whose mothers reported having had medical problems during pregnancy (35.1%) were 1.94 more likely to have incomplete NSCP (*p* = 0.016). The results show a high prevalence of medical problems during pregnancy among mothers of children with NSCP. Evidence about medical problems in pregnancy with the development of NSCP still needs to be well evaluated. Studies in Brazil showed a rate of 21.3% [15] and 76.4% [24] of NSCP children of pregnancies with medical problems, however the subtype of NSCP was not considered.

There is a slight tendency to develop complete NSCP in children with a family history of NSOC (11.2%; OR: 1.44; 0.80–2.55), whose mother used some type of medication during pregnancy (14.0%; OR: 1.35; 0.76–2.37) and present with some kind of systemic alteration (16.5%; OR: 1.21; 0.74–1.97). Even with no statistically significant differences between the groups (*p* > 0,005), it is necessary to discuss these findings in the cleft type development. In our study, 24.5% of patients had family history of oral clefts. This finding is higher than the 3.0% prevalence in Spain [21] and 6.8% in Brazil [17]. Although, in study realized in another region in Brazil, the prevalence of NSOC family history was 33.2% NSCP individuals [15]. It is believed that the results may have been influenced by the sample sizes of their respective studies.

Systemic alterations were identified in 40.6% of the participants. Previous studies show associations of NSCP with other anomalies and congenital alterations [4, 11, 14, 18, 25, 26]. Furthermore, cleft palate only is a predictor of more than 400 different syndromes [10, 19].

Use of medication during the gestational period had no association with any NSCP type, but it was present in 34.3% of the analyzed individuals. Epidemiological case-control study carried out across 15 years in Iran got association between corticosteroids, antiemetics, abortive drugs, barbiturates and anticonvulsant drugs use with the oral clefts [4]. And most specific about NSCP, Jackson et al. [5] identified that exposure to valproic acid was a risk factor to development, because of the folate antagonistic mechanism, which interrupts DNA synthesis leading to congenital malformations [27].

Just under 18% of participants did not undergo orthognathic surgery. This finding is in line with a previous study that concluded that one in eight patients with isolated NSCP requires orthognathic surgery. Being more suitable for Asians and lower in patients of white descent [28].

Dental care is important in the multidisciplinary approach of the cleft patients. We visualize that complete NSCP patients were 2.70 times more likely to have not undergone dental treatment. In addition, these patients were 1.36 times more likely to have not undergone surgical treatment yet. The treatment of complete NSCP presents challenges. Protocols are not standardized yet, but there is a consensus that the first reconstructive surgery should be performed before one year of life [29]. We noticed that only 14.8% of participants underwent surgery at the correct time (< 1 year of life). However, it is important to emphasize that this finding may be influenced by other contextual variables, such as the Brazil size, number of specialized centers and lack of information for patients about seeking treatment as soon as possible.

We recommend that further surveys be conducted in different populations, with paired samples of other types of clefts and control groupsIn order to better identify the etiopathogenesis of this congenital anomaly. As limitations the present research was not conducted in all referral services for NSOC individuals in Brazil. It is interesting to carry out a larger survey about NSCP patients.

## Conclusions

In this study incomplete NSCP was the most prevalent mainly in white skin color female. Medical problems during pregnancy were associated with the complete subtype, being a predictor of the etiogenesis of this anomaly. Even without showing a significant association, medication use during pregnancy and individuals with a positive family history of oral clefts had a higher odds ratio of manifesting complete cleft palate. It should be noted that this is one of the first surveys in more than one Brazilian center to estimate differences in the subtypes of isolated NSCP.

## Data Availability

The datasets used and/or analysed during the current study are available from the corresponding author on reasonable request.

## List of abreviations

NSCL: Nonsyndromic cleft lip
NSCLP: Nonsyndromic cleft lip and palate
NSOP: Nonsyndromic oral cleft
NSCP: Nonsyndromic cleft palate

## References

[1] Dixon MJ, Marazita ML, Beaty TH, Murray JC (2011). Cleft lip and palate: understanding genetic and environmental influences. Nat Rev Genet.

[2] Alade A, Awotoye W, Butali A (2022). Genetic and epigenetic studies in non-syndromic oral clefts. Oral Dis.

[3] Hammond NL, Dixon MJ (2022). Revisiting the embryogenesis of lip and palate development. Oral Dis.

[4] Zandi M, Heidari A (2011). An epidemiologic study of orofacial clefts in Hamedan City, Iran: a 15-year study. Cleft Palate Craniofac J.

[5] Jackson A, Bromley R, Morrow J, Irwin B, Clayton-Smith J (2016). In utero exposure to valproate increases the risk of isolated cleft palate. Arch Dis Child Fetal Neonatal Ed.

[6] Ács L, Bányai D, Nemes B, Nagy K, Ács N, Bánhidy F (2020). Maternal-related factors in the origin of isolated cleft palate-A population-based case-control study. Orthod Craniofac Res.

[7] Raut JR, Simeone RM, Tinker SC, Canfield MA, Day RS, Agopian AJ (2019). Proportion of Orofacial Clefts Attributable to recognized risk factors. Cleft Palate Craniofac J.

[8] Kapos FP, White LA, Schmidt KA, Hawes SE, Starr JR (2021). Risk of non-syndromic orofacial clefts by maternal rural-urban residence and race/ethnicity: a population-based case-control study in Washington State 1989–2014. Paediatr Perinat Epidemiol.

[9] Coutinho ALF, Lima MC, Kitamura MAP, Neto JF, Pereira RM (2009). Epidemiological characteristics of patients with orofacial clefts attending a Referral Center in Northeast Brazil. Rev Bras Saúde Matern Infant.

[10] Mossey PA, Little J, Munger RG, Dixon MJ, Shaw WC (2009). Cleft lip and palate. Lancet.

[11] Jugessur A, Farlie PG, Kilpatrick N (2009). The genetics of isolated orafacial clefts: from genotypes to subphenotypes. Oral Dis.

[12] Silva CM, Pereira MCMP, Queiroz TB, Neves LT (2021). Family history in non-syndromic orofacial clefts: is there a pattern?. Oral Dis.

[13] Cuozzo FDM, Espinosa MM, da Silva KTS, de Barros YBAM, Bandeca MC, Aranha AMF (2013). Cleft lip and palate in a brazilian subpopulation. J Int Oral Health.

[14] Koga H, Iida K, Maeda T, Takahashi M, Fukushima N, Goshi T (2016). Epidemiologic research on Malformations Associated with Cleft Lip and Cleft Palate in Japan. PLoS ONE.

[15] Ferrari-Piloni C, Barros LAN, Jesuíno FAZ, Valladares-Neto J (2021). Prevalence of cleft lip and palate and associated factors in Brazil’s Midwest: a single-center study. Braz Oral Res.

[16] Hlongwa P, Levin J, Rispel LC (2019). Epidemiology and clinical profile of individuals with cleft lip and palate utilizing specialized academic treatment centers in South Africa. PLoS ONE.

[17] Martelli DRB, Machado RA, Swerts MSO, Rodrigues LAM, de Aquino SN, Martelli Júnior H (2012). Non syndromic cleft lip and palate: relationship between sex and clinical extension. Braz J Otorhinolaryngol.

[18] Sarmiento K, Valencia S, Gracia G, Hurtado-Villa P, Zarante I (2018). Clinical and epidemiologic description of Orofacial Clefts in Bogota and Cali, Colombia, 2001–2015. Cleft Palate Craniofac J.

[19] Burg ML, Chai Y, Yao CA, Magee W, Figueiredo JC (2016). Epidemiology, etiology, and treatment of isolated cleft palate. Front Physiol.

[20] Freitas AB, Carvalho CA, Martelli DRB, Barros LM, Bonan PRF, Martelli-Júnior H (2009). Cleft lip and palate: study of a population attended to by a reference service from the state of Minas Gerais. Arq Odontol.

[21] Vico RMY, Linares AI, Mendo IG, Lagares DT, Moles MAG, Pérez JLG (2012). A descriptive epidemiologic study of cleft lip and palate in Spain. Oral Surg Oral Med Oral Pathol Oral Radiol.

[22] Shibukawa BMC, Rissi GP, Higarashi IH, Oliveira RR (2019). Factors associated with the presence of cleft lip and / or cleft palate in brazilian newborns. Rev Bras Saúde Mater Infant.

[23] Machado RA, Toledo IP, Martelli-Júnior H, Reis SR, Guerra ENS, Coletta RD (2018). Potential genetic markers for nonsyndromic oral clefts in the brazilian population: a systematic review and meta-analysis. Birth Defects Res.

[24] Gardenal M, Bastos PRHO, Pontes ERJC, Bogo D (2011). Prevalent diagnosis of Orofacial Fissures in a Reference Service with Resident cases in the state of Mato Grosso do sul. Intl Arch Otorhinolaryngol.

[25] Souza J, Raskin S (2013). Clinical and epidemiological study of orofacial clefts. J Pediatr.

[26] Gil-da-Silva-Lopes VL, Monlléo IL (2014). Risk factors and the prevention of oral clefts. Braz oral res.

[27] Morrell MJ (2002). Folic acid and epilepsy. Epilepsy Curr.

[28] Antonarakis GS, Watts G, Daskalogiannakis J (2015). The need for orthognathic surgery in nonsyndromic patients with repaired isolated cleft palate. Cleft Palate Craniofac J.

[29] Martelli-Júnior H, Porto LV, Martelli DRB, Bonan PRF, Freitas AB, Coletta RD (2007). Prevalence of nonsyndromic oral clefts in a reference hospital in the state of Minas Gerais, Brazil, between 2000–2005. Braz Oral Res.

